# Diagnosis of primary pulmonary T- cell/histiocyte-rich large B cell lymphoma with tissue eosinophilia via clinicopathological observation and molecular assay

**DOI:** 10.1186/s13000-014-0188-6

**Published:** 2014-10-02

**Authors:** Jin Zhu, Yingmei Wang, Li Gong, Gaosheng Huang

**Affiliations:** The State Key Laboratory of Cancer Biology, Department of Pathology, Xijing Hospital, Fourth Military Medical University, Changle West Road #169, Xi’an, 710032 People’s Republic of China; Department of Clinical Laboratory, The Affiliated Hospital of Lintong Sanatorium, Lanzhou Military Command, Qinling North Road, Xi’an, 710600 People’s Republic of China; The Helmholtz Sino-German Laboratory for Cancer Research, Department of Pathology, Tangdu Hospital, the Fourth Military Medical University, Xi’an, 710038 People’s Republic of China

**Keywords:** Primary pulmonary lymphoma, B cell lymphoma, Tissue eosinophilia, Gene rearrangement

## Abstract

**Background:**

Primary pulmonary lymphoma (PPL) is rare and easily misdiagnosed because of the lack of typical clinical features. It most commonly involves elderly patients aged between 60 and 70 years, and pathological diagnosis depends mainly on chest surgery rather than bronchial mucosal biopsy. Via percutaneous needle aspiration biopsy of the lung of a 33-year-old woman, which had distinct tissue eosinophilia, we diagnosed a rare case of rapidly growing large B cell lymphoma.

**Methods:**

Bronchial mucosal biopsy and computed tomography–guided percutaneous needle aspiration biopsy were performed to determine the nature of the lesion, and we identified its immunophenotype using immunohistochemistry. We used BIOMED-2 gene rearrangement PCR to determine lymphocyte clonality; laser microdissection was used to confirm the clonality of suspicious malignant lymphocytes.

**Results:**

Morphologically, the lesion was composed of a large number of eosinophilic cells and a few lymphoid cells. Immunohistochemical staining revealed a few CD1α-positive cells, but they were S-100–negative. The small lymphoid cells predominantly expressed CD3; the large lymphoid cells expressed CD20 and some scattered large lymphoid cells expressed Pax5. However, molecular studies confirmed clonal immunoglobulin heavy chain (IGH)-D gene rearrangement in Pax5–positive large B lymphocytes.

**Conclusions:**

This is the first recorded case of T- cell/histiocyte-rich large B cell lymphoma with tissue eosinophilia of the lung. It highlights the unusual morphological features of PPL that might be mistaken for eosinophilic granuloma or parasitic infection. In addition, IGH and T cell receptor gene rearrangement play important roles in differentiating rare B cell lymphoma from lung space–occupying lesions with abundant eosinophils or T cell infiltration.

**Virtual Slides:**

The virtual slide(s) for this article can be found here: http://med.motic.com/MoticGallery/Slides/AC5C9A6F-46EC-4C71-A448-1312F6900C65?user=2C69F0D6-A478-4A2B-ABF0-BB36763E8025

## Background

Primary pulmonary lymphoma (PPL) is defined as a clonal lymphoid proliferation affecting one or both lungs in a patient with no detectable extrapulmonary involvement at diagnosis. It is a rare entity, representing less than 1% of primary pulmonary malignancies and accounting for 4–11% of extranodal lymphomas [1-3]. Due to its atypical clinical manifestation and complicated imaging features, PPL is easily misdiagnosed and incorrectly treated. Historically, PPL diagnosis mainly depends on chest surgery to obtain pathological evidence, and most cases cannot be diagnosed only by bronchial mucosal biopsy [4,5]. We describe a case of primary pulmonary large B cell lymphoma with marked tissue eosinophilia and increased T cells in which histological, immunohistochemical, and gene rearrangement findings were combined to obtain a diagnosis by computed tomography (CT)-guided percutaneous needle aspiration biopsy.

## Case presentation

### Clinical history

**Figure 1:**
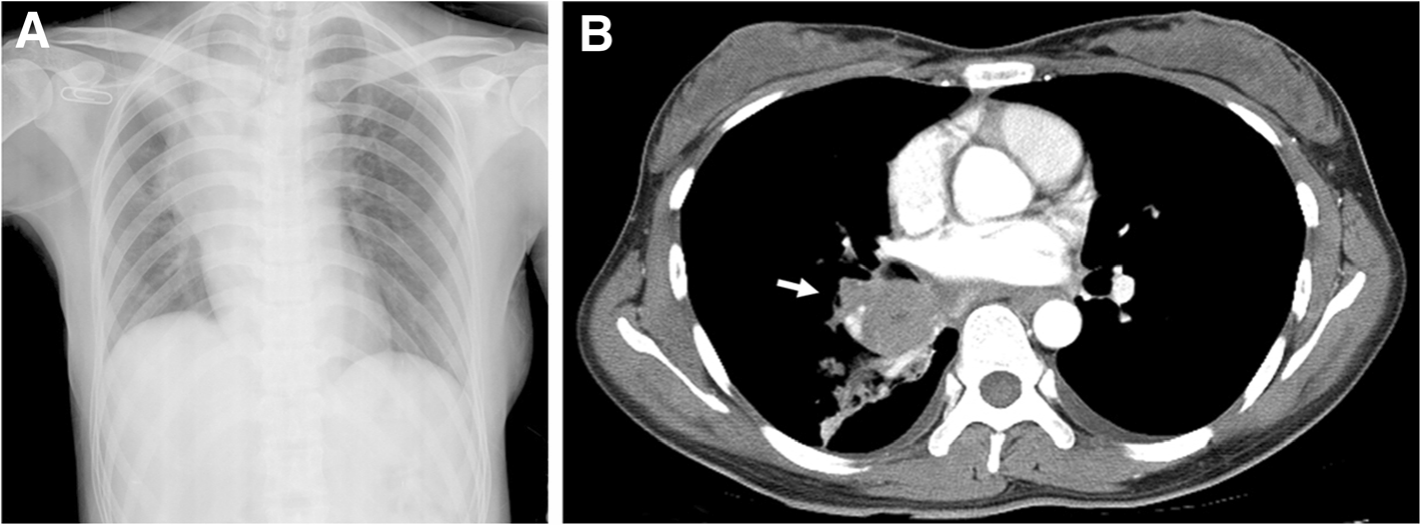
**Chest radiograph and lung CT scan. A**: Chest radiograph showing widened mediastinum in the upper-middle lobe and upper lobe atelectasis in the right lung, indicating a space-occupying lesion. **B**: Lung CT scan showing a right hilar mass measuring about 5.0 × 4.8 cm (arrow).

A 33-year-old woman had cough and expectoration without fever or other discomfort. A peripheral blood count revealed an increased white cell count, but there was no obvious abnormal change on chest radiographs. Thus, the local physician attributed her symptoms to allergic bronchitis and she received anti-inflammatory treatment. However, she visited the hospital again after five months because of the same symptoms, which were accompanied by severe shortness of breath. Laboratory examination revealed increased white blood cells in the peripheral blood, but the absolute value of eosinophilic granulocytes was in the normal range. Other tests showed increased erythrocyte sedimentation rate (ESR), negative autoantibody, carcinoembryonic antigen far lower than the reference value, but a weakly positive Postscript Printer Description skin test. Chest radiography and CT scan revealed a space-occupying lesion in the upper lobe of the right lung, which indicated lung cancer with atelectasis of the right upper lung (Figure 1) without nodal involvement. We performed bronchial mucosal biopsy and CT-guided percutaneous needle aspiration biopsy (aspiration biopsy), which revealed extensive eosinophil infiltration and no evidence of malignancy. Positron emission tomography scan and bone marrow biopsy were also normal. Repeated sputum cultures and parasite antigen testing excluded bacterial and parasitic infection, respectively. In addition, we did not find acid-fast bacilli from smears or bronchoalveolar lavage fluid (BALF), and the *Mycobacterium tuberculosis* (TB) DNA copy number from the BALF and lung biopsy tissues were all <25 copies/mL; therefore, we also excluded TB. The lesion was suspected eosinophilic granuloma, and the patient received an experimental glucocorticoid treatment that successfully alleviated her symptoms. However, subsequent CT scans revealed an increased mass, and the patient underwent biopsy again. While her bone marrow biopsy was normal, the ESR remained increased.

## Methods

### Immunohistochemistry

Immunohistochemical staining was performed using EnVision™ Systems (Dako, Glostrup, Denmark) according to the manufacturer’s instructions. The primary antibodies used were mouse anti-human antibodies against anaplastic lymphoma kinase (ALK), clusterin, CD34, AE1/AE3, CD30, CD1α, CD20, and CD68, and rabbit anti-human antibodies against S-100, CD3, and Pax5. All reagents were supplied by Dako but CD3 and CD34 was from Maixin Biotechnology Corp. Ltd. (Fuzhou, China).

### Laser microdissection

Ten 10-μm tissue sections (1.5 × 1.5 cm^2^) were obtained from representative paraffin blocks and placed on an ultraviolet (UV)-absorbing membrane. The sections underwent laser microdissection using an LMD6000 laser microdissection microscope (Leica Microsystems Ltd., Wetzlar, Germany). After immunohistochemical staining, slides were mounted on a microstat, and Pax5–positive lymphoid cells were dissected using a UV laser in motorized optical beam scanning mode. The membrane (with the attached specimen) was dropped into the cap of a 0.5-mL microcentrifuge tube and prepared for DNA extraction. For each dissected lesion, a similar volume of surrounding normal lung tissue was isolated and analyzed as a control.

### DNA extraction

Four 20-μm thick sections from formalin-fixed, paraffin-embedded (FFPE) tissue blocks obtained by aspiration biopsy were cut into centrifuge tubes. We isolated DNA from microdissection samples or aspiration biopsy samples using the RecoverAll™ Kit (AM1975; Ambion, Foster City, CA, USA) according to the manufacturer’s protocol. Briefly, FFPE tissue samples were deparaffinized with xylene and graded ethanol and then digested with the appropriate volume of lysate buffer (40 μL for microdissection samples; 100 μL for aspiration biopsy samples) and proteinase K (2 μL for microdissection samples; 6 μL for aspiration biopsy samples) in a water bath (50 °C) for 16 h. We added the appropriate volume of isolation additive/ethanol mixture to each sample and filtered or washed them repeatedly. After RNase digestion and DNA purification, the genomic DNA was extracted and stored at −20°C.

### BIOMED-2 gene rearrangement analysis

We evaluated the clonality of T cell receptor (TCR) and immunoglobulin heavy chain (IGH) genes using the PCR technique described by Liu et al. [6]. The reaction conditions were selected for a final volume of 50 μL containing 100 ng DNA, 200 μM dNTP, 10 pmol each primer irrespective of total numbers of primers in each multiplex PCR tube, 1.5 mM MgCl_2_, and 1 U *Taq* enzyme. Amplification was performed using an Eppendorf thermal cycler (Eppendorf, Hamburg, Germany) for 35 cycles (denaturation at 94°C for 1 min, annealing at 60°C for 1 min, extension at 72°C for 1 min). The PCR products were visualized using polyacrylamide gel electrophoresis.

## Results

### Pathological observation

**Figure 2:**
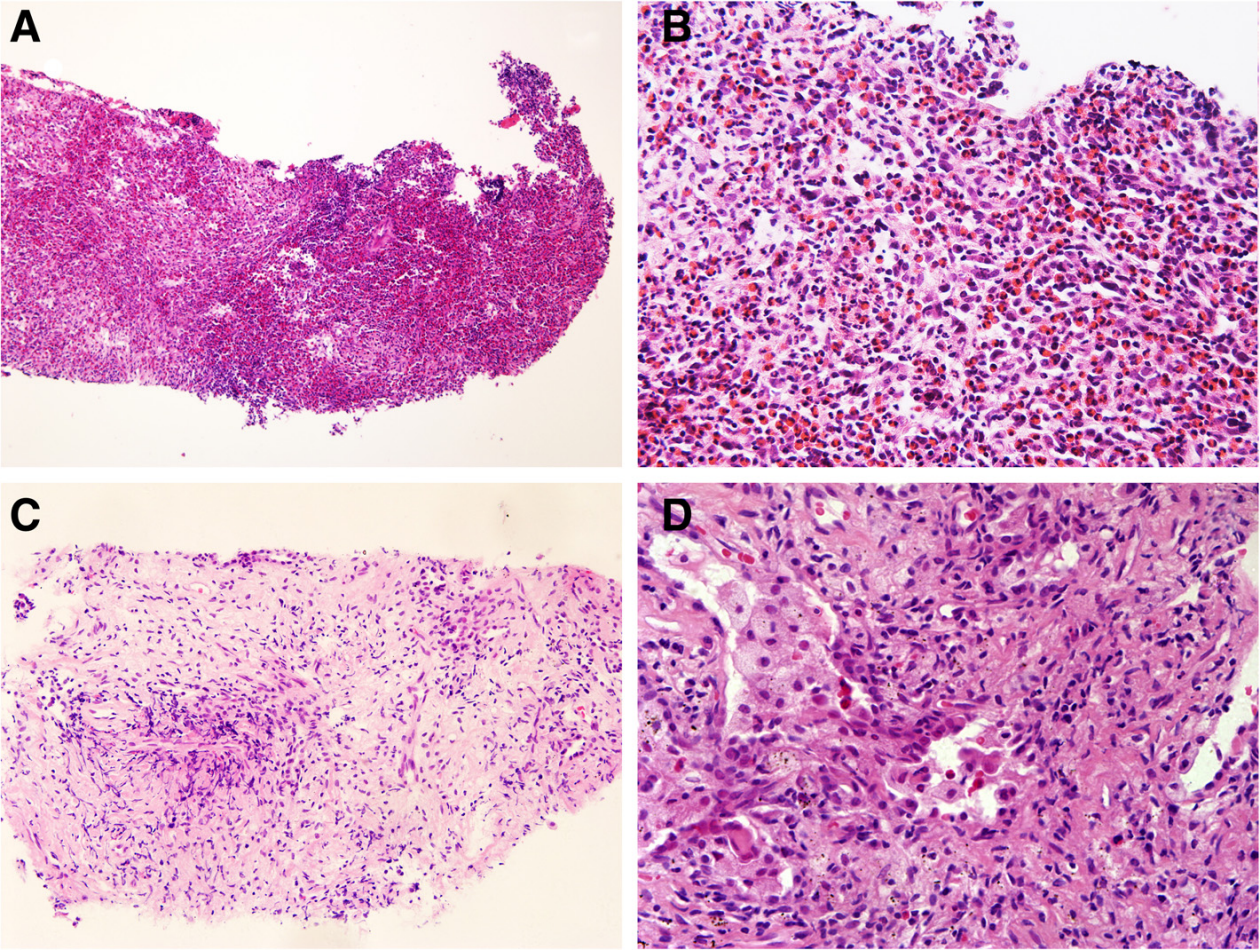
**Histopathological characteristics of the lesion. A**, **B**: Hematoxylin–eosin (H&E)-stained aspiration biopsy section showing marked eosinophilia in the lesion (Figure 2A, ×100; Figure 2B, ×400). **C**, **D**: H&E-stained aspiration biopsy after glucocorticoid treatment showing only a few eosinophils (Figure 2C, ×200; Figure 2D, ×400).

**Figure 3:**
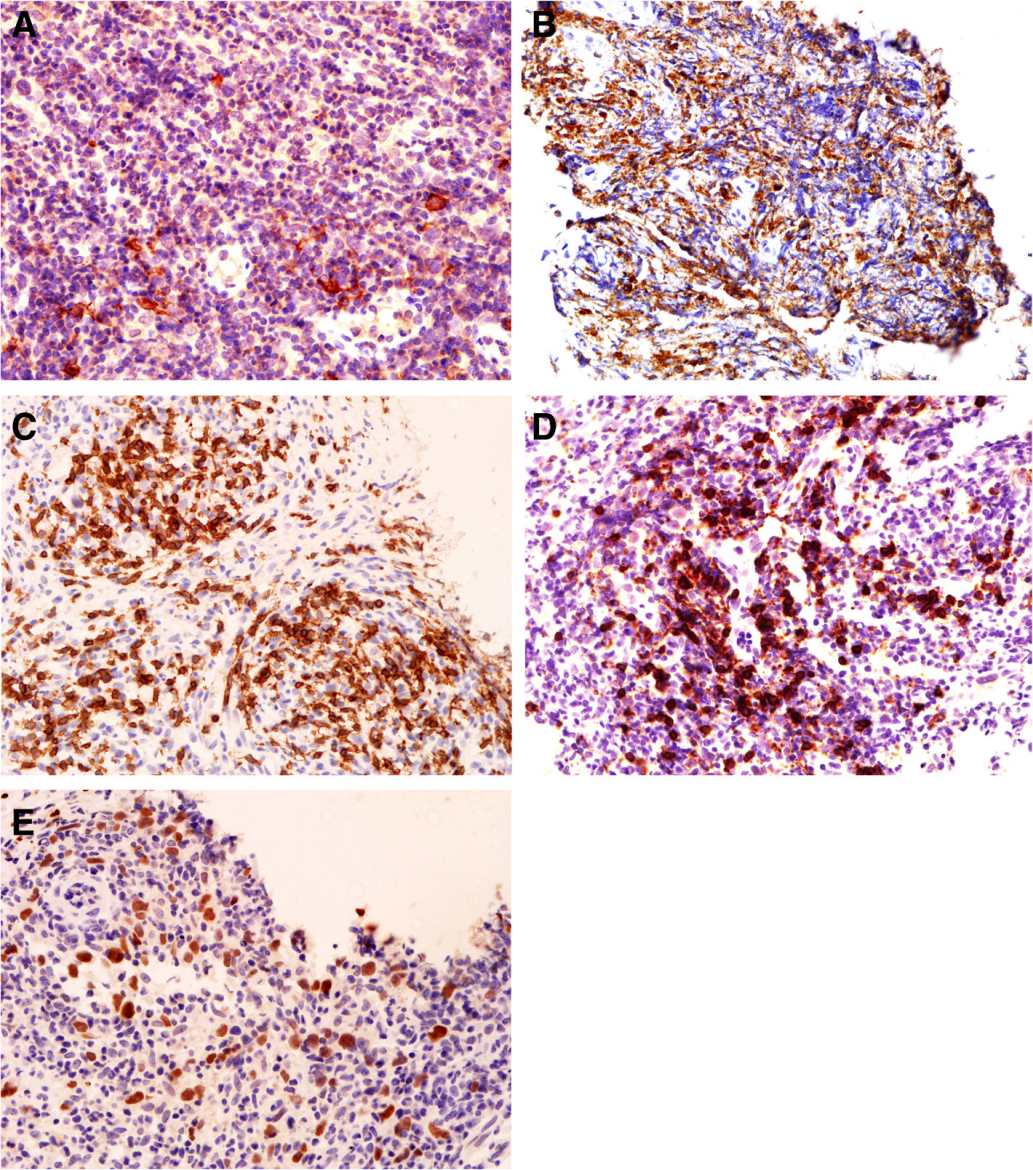
**Immunohistochemical features of the lesion. A**: Immunohistochemical CD1α staining showing only a few CD1α-positive cells (×400). **B**: Immunohistochemical CD68 staining revealing a diffused pattern of CD68-positive cells (×400). **C**: Immunohistochemical CD3 staining revealing a predominant nodular pattern of CD3-positive cells (×400). **D**: Immunohistochemical CD20 staining revealed that many large lymphoid cells were positive. **E**: Immunohistochemical Pax5 staining showing scattered, Pax5–positive large B cells (×400).

At the first aspiration biopsy, histopathology revealed numerous eosinophilic cells infiltrating the lesion (Figure 2A-B), and no definite tumor cells were found. Immunohistochemical staining was negative for S-100, ALK, clusterin, CD34, AE1/AE3; there were only a few large CD30-positive and CD1α-positive cells (Figure 3A), but more CD68-positive cells (Figure 3B).

Following glucocorticoid treatment, histopathology revealed focal accumulation of lymphocytes and less eosinophil infiltration (Figure 2C-D). Immunohistochemical staining showed that the background small lymphocytes predominantly expressed CD3 (Figure 3C), while many large lymphoid cells strongly expressed CD20 (Figure 3D) and some large lymphocytes expressed Pax5 (Figure 3E), and their average nuclei diameter was approximately 11 μm. The distribution of Ki67-positive cells was not even, but most of the cells were scattered larger cells.

### Gene rearrangement

**Figure 4:**
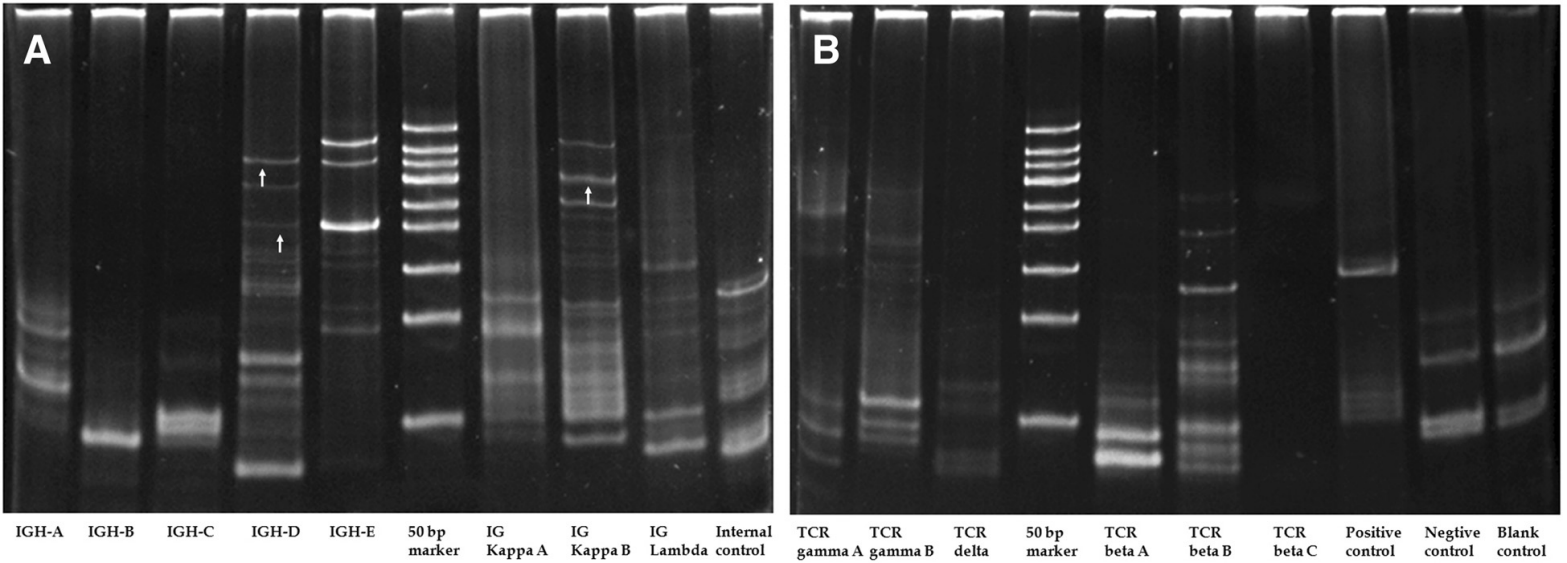
**Results of gene rearrangement analysis of aspiration biopsy tissue. A**: IGH gene rearrangement analysis revealing rearrangement of clonal genes, including IGH-D (arrow) and IGK-B (arrow). Lane 1: IGH-A; lane 2: IGH-B; lane 3: IGH-C; lane 4: IGH-D; lane 5: IGH-E; lane 6: 50-bp marker; lane 7: IGK-A; lane 8: IGK-B; lane 9: IG lambda (L); lane 10: internal control. **B**: TCR gene rearrangement showing no band and no evidence of clonal gene rearrangement. Lane 1: TCR-gamma(G)A; lane 2: TCR-GB; lane 3: TCR-delta(D); lane 4: 50-bp marker; lane 5: TCR-beta(B)A; lane 6: TCR-BB; lane 7: TCR-BC; lane 8: positive control; lane 9: negative control; lane 10: blank control.

**Figure 5:**
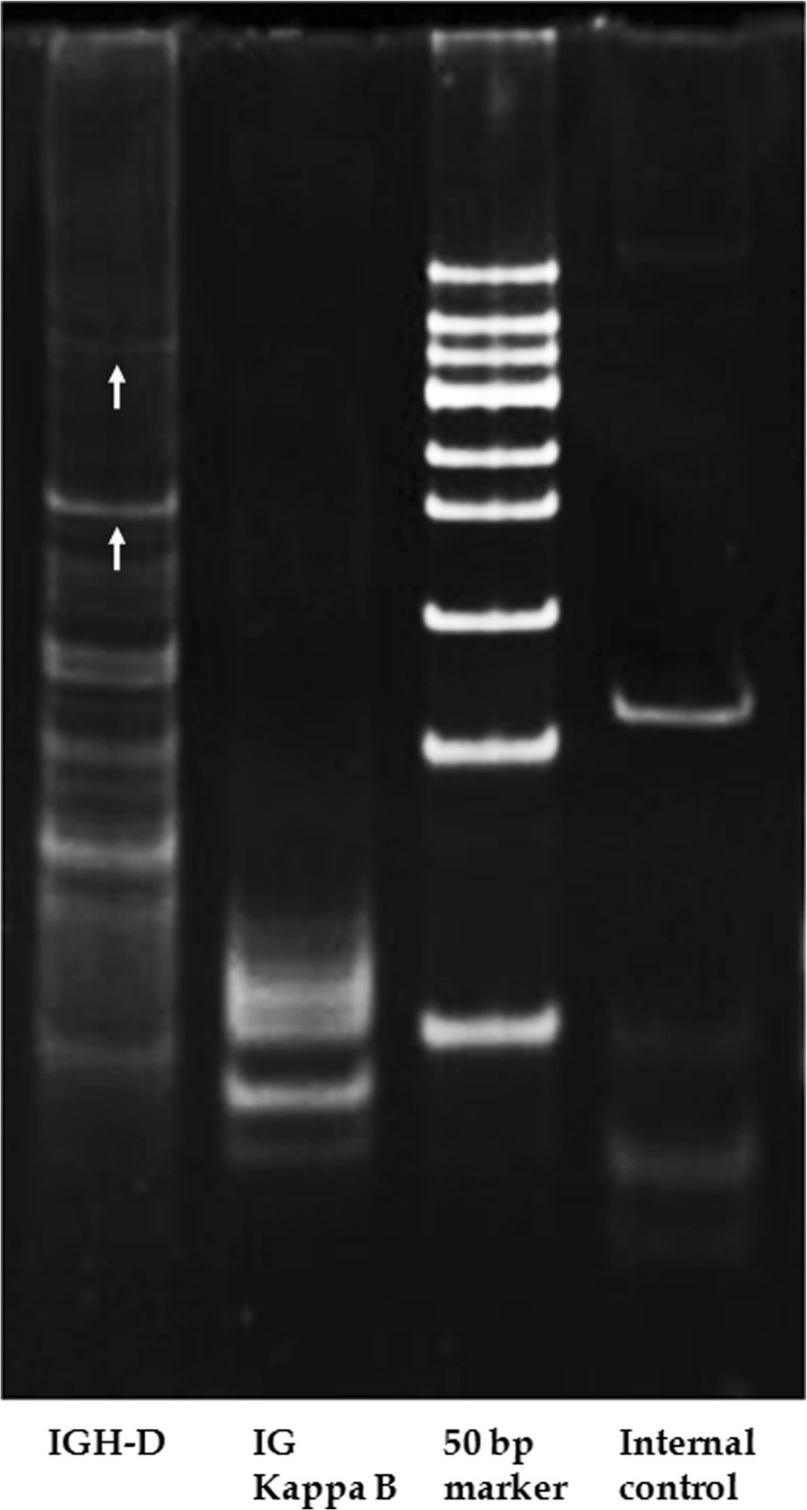
**Results of gene rearrangement analysis of microdissection tissue.** Laser capture microdissection of Pax5–positive large B lymphocytes confirm clonal IGH-D (arrow) gene rearrangement. Lane 1: IGH-D; lane 2: IGK-B; lane 3: 50-bp marker; lane 4: internal control.

Gene rearrangement analysis of the lung biopsy tissue revealed rearrangement of clonal IGH genes, including IGH-D and IG kappa (K)-B, and no clonal rearrangement of TCR genes (Figure 4). Laser capture microdissection determined that the IGH-D gene rearrangement (Figure 5) mainly occurred in Pax5–positive large B lymphocytes.

### Diagnosis and outcome

Based on the histopathological characteristics, immunohistochemical features, and gene rearrangement analysis, we eventually diagnosed the patient with primary pulmonary T- cell/histiocyte-rich large B cell lymphoma with tissue eosinophilia. Subsequently, she completed three cycles of chemotherapy dominated by the cyclophosphamide, doxorubicin, vincristine, and prednisone (CHOP) or CHOP-like regimen. CT scan performed at the last follow-up revealed a significantly reduced mass.

## Discussion

PPL is very rare, and its biological features, clinical presentation, prognostic markers, and treatment are not well defined. Although PPL has various clinical symptoms, they are nonspecific and contribute little to the diagnosis. Furthermore, 37.5–50% of patients are asymptomatic [2,7]. Moreover, the rate of constitutional symptoms and acute-phase reactants (sedimentation, C-reactive protein) are high in patients with PPL, which is also not specific to the diagnosis [8]. Thus, in addition to the pathological manifestations of bronchial mucosal biopsy and surgical biopsy, abnormal chest radiography and CT images may also be of value for diagnosis. Recently, increased numbers of disuse large B-cell lymphoma (DLBCL) cases with various manifestations have been reported, which could be misdiagnosed as TB [9] or lung cancer [10]. A long-term follow-up study of PPL-DLBCL, the largest known series thus far, noted strong homogeneity among the patients: low clinical risk, early stage, and no bulky tumor mass, normal lactic dehydrogenase and beta 2 microglobulin [11]. Yet, some cases also have unusual morphological features, where primary pulmonary B cell lymphoma might present as a necrotic mass or mediastinal type [12,13].

The most common histopathological subtypes of PPL are mucosa-associated lymphoid tissue (MALT) lymphoma and diffuse large B cell lymphoma (DLBCL), which occurs only in 10% of cases [14,15]. In general, there is no clear classification of DLBCL encountered in pulmonary systems. In fact, PLL-DLBCL has been reported only in case reports [9,10], most subtypes of them are centroblastic or immunoblastic variants, as elsewhere, which are the more common morphological variants of DLBCL [1,11,16,17]. In addition, some rare types of DLBCL, such as the anaplastic variant, were also reported [18]. Histopathologically, the case we presented here contained both large and small lymphoid cells, and abundant eosinophils were also found in the tumor tissue. Immunohistochemical staining revealed that the large lymphoid cells were B-cells (both CD20- and Pax5-positive) and small lymphoid cells were predominantly T-cells (CD3-positive) in nature. Our finding is obviously different from the studies [11,13,19] in which immunophenotype of primary B cell lymphoma of the lung is commonly CD3-negative. Meanwhile, many CD68-positive cells were also found, but there were only a few large CD30-positive and CD1α-positive cells in the tumor tissue. In addition, no any S100-positive and definite CD15-positive cells were found. It means that large B-cells are mingled with abundant small T-cells and histiocytes. BIOMED-2 gene rearrangement analysis [6] of both the lung tissue and tumor cells confirmed that large B-cells are neoplastic and small T-cells are reactive. Therefore, the diagnosis of this case is T- cell/histiocyte-rich large B cell lymphoma (THRLBCL), which is a special and rare subtype of DLBCL. Based on the typical cellular and immunohistochemical features of THRLBCL, which is composed of the dispersed large B-cells, predominant small T-cells and more histiocytes, it is not difficult to differentially diagnose it from other large B-cell lymphoma. If there is a difficult case, laboratory evaluation for EBV [20,21] and immunohistochemical staining of OCT2 and BOB1 [22,23] may be useful for the differential diagnosis of THRLBCL with other large B-cell lymphoma.

Upon histopathological examination, a marked increase of eosinophils in non-blood tissue could be diagnosed as tissue eosinophilia. However, eosinophilia refers to increased eosinophils (>0.45 × 10^9^/L) in the peripheral blood [24]. Peripheral blood and tissue eosinophilia may occur in many disorders, including allergic and hypersensitivity diseases, parasitic and viral infections, atopic reactions, immune complex disorders, connective tissue diseases, and malignant tumors. Both solid tumors and hematopoietic neoplasms are associated with eosinophilia. Eosinophilia and tissue eosinophilia are frequent findings in Hodgkin lymphoma [25] and are sometimes observed in patients with lymphoma, predominantly T cell lymphoma or occasionally anaplastic large cell lymphoma [26,27]. Historically, large B cell lymphoma with marked eosinophilia is rare, and only a few cases of mediastinal/thymic large B cell lymphoma may present a few reactive infiltrating lymphocytes and eosinophils. Its clinical manifestation is an anterior mediastinal space-occupying lesion, usually accompanied by superior vena cava syndrome, and is easily misdiagnosed as Hodgkin lymphoma. To date, there are only a few reports on eosinophilia or tissue eosinophilia in B cell lymphoma, and all biopsy samples are obtained from lymph nodes [28-30]. In our case, however, it was primary pulmonary B cell lymphoma without evidence of nodal involvement, and all biopsy samples were obtained from lung tissue. The peripheral blood eosinophilic granulocyte count was in the normal range, and the bone marrow biopsy was normal. These findings did not support the diagnosis of eosinophilia. However, histopathological examination disclosed a lesion with marked eosinophil infiltration, some of which formed eosinophilic abscesses.

The differential diagnoses of eosinophil-rich lesions of the lung included eosinophilic granuloma, Hodgkin’s lymphoma and parasitic or viral infections. In eosinophilic granuloma, there are a large number of characteristic Langerhans cells, which have typical nuclear grooves and showed both CD1α and S100 diffuse positive staining. Furthermore, the gene rearrangement analysis can not reveal the clonal rearrangement of IGH genes. In classical Hodgkin’s lymphoma, there are the typical Reed-Sternberg cells and their variants. In addition, abundant CD30- and CD15- positive cells may be also helpful for differential diagnosis with a large B-cell lymphoma. In parasitic or viral infections, it would yield polypides, eggs, or viral inclusion bodies under microscopy. Taken together, eosinophilic granuloma, Hodgkin’s lymphoma and parasitic or viral infections could be excluded respectively.

Based on the cellular, immunohistochemical and molecular findings, we think that this is a unique case of primary pulmonary large B cell lymphoma with tissue eosinophilia.

The mechanism clarifying the relationship between tissue eosinophilia and B cell lymphoma remains unknown. Typically, it is believed that the eosinophilia is caused by the production of various cytokines, such as interleukin (IL)-5, IL-3, and granulocyte–macrophage colony–stimulating factor (GM-CSF) by B cell lymphoma cells or non-neoplastic T lymphocytes activated by B cell lymphoma cells [30,31]. In addition to the eosinophil chemotactic factors IL-3 and GM-CSF, IL-5 is a major soluble factor for mediating eosinophilia. It can be both a paracrine secretion from T cells [32] and an autonomous autocrine secretion from activated eosinophils [33,34]. Although the role of IL-4 in eosinophil recruitment is controversial [31], IL-4 is highly expressed by T cytotoxic 2 cells, with abundant background eosinophils, in CD8^+^ lymphomatoid papulosis [35]. Generally, these secreted cytokines may either act locally, recruiting eosinophils to the neoplasm, or have a systematic effect, increasing the number of eosinophils maturing and leaving the bone marrow [29]. Although the reason for tissue eosinophilia is as yet unknown, a large study of 1511 diagnostic biopsy specimens of patients has revealed that tissue eosinophilia correlated strongly with poor prognosis in nodular sclerosing Hodgkin’s disease [36]. Similarly, the prognosis of large B cell lymphoma with tissue eosinophilia remains unknown for the rare of such cases and should also be considered.

There is evidence that MALT lymphoma of the lung is a very indolent disease with the potential for spontaneous regression [37] and that DLBCL of the lung may not portend as poor a prognosis as in other extranodal sites [11]. Prompt pathological diagnosis and initiation of therapy, including surgery, chemotherapy, radiotherapy, or combined treatments, are crucial to patient outcome. To date, the most effective therapy is anthracycline-containing chemotherapy, such as the CHOP or CHOP-like regimen, which is more commonly offered. The long-term follow-up study of Neri et al. showed that patients treated with CHOP achieved complete response (CR) >80% and progression-free survival (PFS) and overall survival (OS) >5 years [11]. Although the addition of rituximab to CHOP (R-CHOP) increased the CR rate, PFS, and OS in DLBCL [38,39], especially in younger patients with low clinical risk, a recent study reported that there was no statistical difference in PPL-DLBCL following a retrospective comparison of patients who received R-CHOP or CHOP alone [40].

## Conclusion

In conclusion, this is the first reported case of T- cell/histiocyte-rich large B cell lymphoma with tissue eosinophilia of the lung, which is difficult to diagnose and is easily confused with other diseases. Given the low incidence and favorable prognosis of PPL B cell lymphoma, a high index of suspicion must be maintained and the diagnosis should be performed with care using immunohistochemical staining; molecular analysis might aid the diagnosis, particularly in the case of small specimens such as that obtained by needle biopsy.

## Consent

Written informed consent was obtained from the patient for publication of this Case Report and any accompanying images.

## Abbreviations

PPL: Primary pulmonary lymphoma
IGH: Immunoglobulin heavy chain
CT: Computed tomography
ESR: Erythrocyte sedimentation rate
BALF: Bronchoalveolar lavage fluid
TB: *Mycobacterium tuberculosis*
UV: Ultraviolet
FFPE: Formalin-fixed paraffin-embedded
TCR: T cell receptor
PCR: Polymerase chain reaction
CHOP: Cyclophosphamide, doxorubicin, vincristine, and prednisone
DLBCL: Disuse large B-cell lymphoma
MALT: Mucosa-associated lymphoid tissue
THRLBCL: T- cell/histiocyte-rich large B cell lymphoma
EBV: Epstein barr virus
IL: Interleukin
GM-CSF: Granulocyte–macrophage colony–stimulating factor
CR: Complete response
PFS: Progression-free survival
OS: Overall survival
